# Refined Separation Treatment for Reclaimed Asphalt Pavement (RAP) Processing: A Review

**DOI:** 10.3390/ma19143050

**Published:** 2026-07-15

**Authors:** Hui Liao, Yang Zhang, Tao Ma, Zhaoqing Chen, Conglin Chen

**Affiliations:** School of Transportation, Southeast University, No.2 Southeast University Road, Nanjing 211189, China; liaohui@seu.edu.cn (H.L.);

**Keywords:** reclaimed asphalt pavement (RAP), variability, RAP agglomeration, refined separation, rotary centrifugal decomposition

## Abstract

The reuse of reclaimed asphalt pavement (RAP) offers economic and environmental benefits in road construction. However, its inconsistent quality—stemming from material complexity and poor management—limits its efficient and high-value application. A key factor contributing to RAP variability is particle agglomeration, where aged asphalt mortar binds aggregate together, leading to inaccurate aggregate gradation measurements and unpredictable contributions of aged binder. These inconsistencies reduce the uniformity and mechanical performance of recycled asphalt mixes. This paper provides a critical review of refined RAP separation treatments recently developed as supplementary RAP processing techniques to reduce variability and enhance performance consistency. Among these methods, rotary centrifugal decomposition has emerged as a promising technique for disaggregating RAP agglomerates and recovering coarse aggregates with minimal residual aged asphalt. Large-scale applications have demonstrated its efficiency in producing well-graded RAP fractions, typically separating recycled coarse aggregates (5–20 mm) from bitumen-rich fine RAP (0–5 mm). Despite its potential to improve RAP recycling rates and high-RAP-content mix performance, challenges remain in preserving coarse aggregate integrity, controlling gradation refinement, and managing the bitumen-rich fine fractions. Therefore, further research is needed to optimize the separation process to minimize material degradation and excessive fine fractions and assess long-term practicality and cost effectiveness through life-cycle analysis.

## 1. Introduction

Reclaimed asphalt pavement (RAP)—milled asphalt pavement consisting of aged asphalt mortar and stone aggregates—is widely used as a sustainable alternative to virgin materials in road infrastructures. The reuse of RAP brings both economic and environmental benefits. It reduces the cost of raw materials, lowers Greenhouse Gas (GHG) emissions from production and transportation of virgin materials, conserves non-renewable bituminous binders and well-graded aggregates, and reduces landfill demand for construction waste [1,2,3,4,5,6]. Despite these advantages, current recycling strategies cannot handle the vast quantities of RAP generated annually from pavement rehabilitation and reconstruction. Prolonged RAP storage stiffens and embrittles the aged binder, increases stockpile management costs, and induces soil and groundwater contamination [7,8,9].

A major challenge that limits high-value RAP recycling is material variability. RAP exhibits fluctuations in asphalt content, differences in RAP binder aging, particle agglomeration, and inaccurate gradation. These factors collectively reduce the homogeneity and performance of recycled asphalt mixes [10,11]. Consequently, transportation agencies have established specifications limiting the allowable RAP content in recycled asphalt mixes; contractors remain reluctant to produce high-RAP-content mixes due to concerns over premature pavement failures and durability issues.

In response to these challenges, numerous studies have explored strategies to increase RAP recycling rates, including improved RAP processing and management practices [12], using warm-mix asphalt additives and rejuvenators to enhance compatibility and blending [13,14,15], and optimizing mix design methodologies for high-RAP-content mixes [16,17]. Although these approaches have shown promise, challenges remain in addressing RAP variability at its source. Recently, refined asphalt–aggregate separation has emerged as a promising RAP processing technique. Unlike conventional crushing and screening, refined separation aims to disaggregate RAP agglomerations and separate aged asphalt mortar from coarse aggregates. Research indicates that this process can effectively isolate coarse RAP aggregates, covered with minimal aged asphalt, from bitumen-rich asphalt mortar with a high asphalt-to-aggregate ratio [18]. Among various separation techniques, rotary centrifugal decomposition has proven efficient at dividing RAP into distinct fractions, and the resulting coarse aggregates can potentially replace virgin aggregates [19,20].

Nonetheless, refined mechanical separation presents several challenges. For instance, it may change RAP gradation and potentially impair surface texture, angularity, and resistance to fragmentation of RAP aggregates. Also, a residual film of aged binder still remains on the surface of separated aggregates, potentially affecting adhesive bonding within asphalt mixes. Unlike the “black rock” situation—where aggregates are fully coated with aged asphalt—refined separation yields partially coated aggregates [21]. It remains unclear whether the partial coating improves adhesion due to chemical compatibility or creates a weak interlayer between RAP aggregates and virgin materials [22,23]. Another challenge pertains to the utilization of the bitumen-rich mortar fraction, which can comprise over 50% of the separated materials. Despite severe aging, this fraction retains a substantial amount of valuable binder.

**Figure 1:**
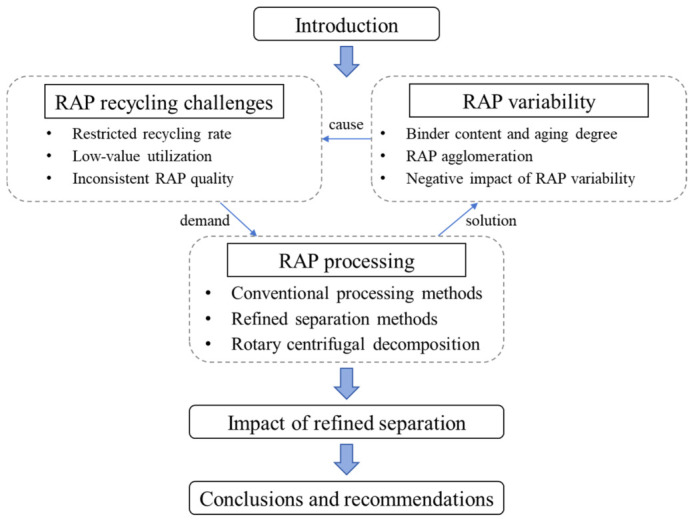
Schematic diagram of review structure.

This review synthesizes developments and applications of refined separation treatments for RAP, focusing on its influence on RAP variability, homogeneity of recycled asphalt mixes, and performance of high-RAP-content mixes. This paper also explores potential pathways for utilizing the separated fine fraction, offering insights into improving the sustainability and efficiency of RAP recycling practices. The novelty of this review lies in its explicit focus on variability reduction as the key performance indicator, providing a structured analysis that links separation efficiency directly to mix performance. The scope is limited to mechanical refined separation methods (especially the centrifugal decomposition) that have been tested at a laboratory or pilot scale. By synthesizing current knowledge, this review offers insights for researchers and practitioners seeking to overcome RAP variability and advance more sustainable high-RAP recycling practices. Figure 1 outlines a schematic diagram to illustrate the review structure.

## 2. Challenges in RAP Recycling

RAP recycling techniques are categorized by treatment location (in-place vs. in-plant) or by processing temperature (hot, warm, or cold mixing) [24,25,26]. In Hot Central Plant Recycling (HCPR), superheated virgin aggregates reactivate the aged RAP binder, facilitating blending between aged and virgin binders [27]. However, HCPR requires significantly more energy than conventional hot-mix production due to the need to superheat virgin aggregates and, in some cases, preheat RAP [28]. Also, RAP binder may undergo secondary aging or carbonization, leading to poor asphalt regeneration and overestimation of binder contribution [29,30]. Warm-mix recycling offers notable energy savings but faces challenges such as gradation variability and poor adhesion between RAP and virgin asphalt due to lower production temperatures. Likewise, in cold-mix recycling, RAP is often treated as a granular material or filler, referred to as “black rock”. The weak bonding between RAP and virgin asphalt limits its use in high-grade pavements [31].

Although these RAP recycling techniques are well established, challenges persist in increasing allowable RAP proportion and maximizing its utilization value. Moreover, RAP is a heterogeneous material composed of components from various projects, road sections, and pavement layers, making quality control difficult under current recycling strategies. This section elucidates the current challenges exist in RAP recycling.

### 2.1. Restricted Recycling Rate

Theoretically, RAP is 100% recyclable, as its major components are aged asphalt binders and high-quality mineral aggregates [32]. Yet, practical limitations restrict the recycling rate, undermining the potential for circular recycling in pavement infrastructure. Studies have shown that recycled asphalt mixes containing up to 30% RAP can perform comparably to virgin asphalt mixes [33,34]. Incorporation of more than 30% RAP often leads to increased mix stiffness and insufficient binder blending, causing premature pavement failure [35,36].

Consequently, most agencies and contractors are hesitant to exceed current RAP content thresholds. For instance, the Ontario provincial standard limits RAP content to 15% in surface layers and 30% in binder layers [37]. Similarly, the Superpave mix design specification recommends lowering the virgin binder performance grade (PG) by one when RAP content exceeds 15% and basing the PG on RAP binder properties when exceeds 25% [38]. In China, the national technical specification no longer imposes a unified threshold. Instead, the allowable RAP percentage is determined by RAP quality, virgin asphalt grade, mixing plant capabilities, and mix type [39]. Table 1 provides guidelines for selecting virgin asphalt grades based on RAP content and aging level.

### 2.2. Low-Value Utilization

Current RAP recycling practices often fail to maximize the value of RAP. Traditional approaches simply focus on replacing virgin materials with RAP, leading to several inefficiencies:

- Failed horizontal recycling: High-quality RAP generated from upper pavement layers is often downcycled in lower layers or base courses.
- Limited regeneration of aged binder: Fine RAP fractions with a higher binder ratio are particularly challenging to regenerate.
- Lack of diversified recycling scenarios: RAP is not always reused according to its unique characteristics, limiting its potential application scenarios.

In binder or base layers, the allowed RAP proportion can reach 50% to 60%, while its use in surface layers is often restricted. In the US, the maximum allowable RAP content in surface layers ranges from 0% to 25%, with only Virginia permitting up to 30% [40]. In China, when RAP content exceeds 30% in surface layers, contractors must conduct thorough demonstrations to justify its use [39].

RAP retrieved from upper pavement layers typically contains high-quality stone aggregates and polymer-modified binders. Incorporating these materials into lower pavement layers, or even into unbound or cement/bitumen-treated base and subbase courses as granular materials, significantly reduces their value. Moreover, current recycling practices fail to fully restore the non-renewable asphalt binder in RAP due to severe aging, especially in fine fractions.

### 2.3. Inconsistent RAP Quality

During pavement service life, asphalt binder undergoes oxidation, volatilization, and aggregate absorption, resulting in increased stiffness and brittleness. Furthermore, aggregate quality declines due to particle pulverization and gradation refinement caused by traffic loading. RAP quality is also influenced by milling operations, including milling speed and depth. Appropriate milling speed can prevent excessive pulverization while maintaining efficiency, whereas milling depth is critical for obtaining homogeneous material by excavating a single asphalt layer [41]. After milling, RAP is stored in mixing plants before reuse. However, limited storage space forces remixing and homogenization of RAP from different sources, making consistent quality difficult to achieve.

RAP management is essential to producing high-quality and consistent materials. Contractors often follow their specific RAP management systems, leading to variability across different plants. For example, fractionation is not always required by regional standards, and the number of fractioned stockpiles is not strictly regulated [42]. Improper stockpiling can cause segregation, consolidation, and moisture retention [41]. The presence of asphalt binder also increases the likelihood of particle agglomeration and the formation of oversized chunks.

In conclusion, the primary challenge in RAP recycling is improving its quality and consistency to enable its use in high-grade road infrastructure. Higher RAP contents frequently lead to increased mix stiffness, inadequate binder blending, and premature distress, which has prompted most specifications to impose conservative thresholds. Consequently, current practices often result in low-value utilization of RAP. Moreover, inconsistent RAP quality—stemming from aging, milling operations, stockpiling, and the blending of multiple sources—undermines quality control and mix reliability. Given that the aging degree of the binder and the inherent characteristics of the aggregates cannot be controlled during processing, the most viable approach is to optimize RAP processing techniques and management practices. By doing so, RAP can serve as a ready-to-use raw material in asphalt mix production, requiring only minimal modifications to existing mix design and recycling technologies.

## 3. RAP Variability

RAP variability arises from the blending of milled pavement materials collected from different project locations and pavement layer positions, which have experienced varied service durations, contamination levels, and milling operations [43,44]. Compared to virgin materials, RAP exhibits substantial variability in both asphalt binder properties and aggregate gradation [45]. One of the most critical contributors to RAP heterogeneity is particle agglomeration: the formation of clusters bonded by aged asphalt mortar [46]. As RAP content increases, the performance of recycled asphalt mixes becomes increasingly dependent on RAP properties. A higher concentration of agglomeration exacerbates deviations in sieve passing rates and causes differential activation of the aged binder. These issues can disrupt gradation design and the asphalt–aggregate ratio, leading to uneven aggregate distribution, insufficient lubrication, and weak adhesive bonding [47].

This section summarizes findings on the characterization of RAP variability, focusing on aged binder properties and particle agglomeration phenomenon, and discusses the impact on recycled asphalt mix performance, especially at high RAP content.

### 3.1. RAP Binder Distribution and Aging Degree

The distribution and aging degree of RAP binder significantly influences the performance of recycled asphalt mixes. Key factors include binder content, differential aging, and the activation and migration of aged binders.

Aged asphalt content in RAP increases as particle size decreases, primarily because finer aggregates have a higher specific surface area and thus absorb more asphalt [48]. Determining the accurate RAP binder contribution is crucial for calculating the appropriate amount of virgin asphalt. The proportion of fines passing the 0.075 mm sieve varies significantly, influencing the binding properties and durability of asphalt mixes [43]. Since fines contribute to the mixture’s texture and cohesion, their variability is a critical consideration in the mix design.

The aging degree of RAP binder varies depending on their origin within the pavement structure. Binders from the upper course are stiffer than those from the binder and base courses due to greater exposure to traffic, moisture, ultraviolet radiation, and oxidation [49]. Saliani et al. [50] found that asphalt binders recovered from different RAP size fractions exhibit varying degrees of hardening. For instance, binders from fine fractions are stiffer and more temperature sensitive than those from coarse fractions. In addition, the outermost surface is more prone to oxidation and is more aged than the internal binder film [51]. Overall, differential aging is influenced more by binder aging level than by particle size. In severely aged RAP, the contrast between the outer and inner binder layers becomes more pronounced [52].

Uneven distribution of RAP binder affects its activation and migration. When using fractionated RAP, aged binder activation depends on differences in binder content and aging levels between coarse and fine RAP fractions [50]. Particle size distribution also influences the interaction between aged and virgin binders, as finer particles offer greater surface area for interaction with virgin binders. Stimilli et al. [53] proposed a revised surface area calculation for RAP to account for the high proportion of fines. However, the agglomeration phenomenon often leads to a lower actual surface area than the theoretical assumption of uniformly dispersed particles, causing overestimation of binder contribution [54]. Therefore, a better understanding of agglomeration breakdown and the formation of new agglomerates is essential for accurately assessing RAP binder availability and migration.

### 3.2. RAP Agglomeration

**Figure 2:**
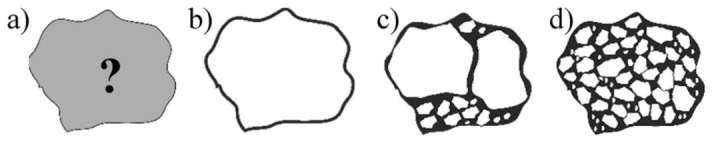
Possible forms of a milled RAP piece: (**a**) unknown internal structure; (**b**) single aggregate; (**c**) coarse aggregate bonded with fines; (**d**) coarse aggregate formed by clustered fines (reused from [57] under the CC BY license, © 2021 The Authors).

RAP variability is primarily driven by agglomeration, which produces pseudo particle sizes and false gradation. After milling from old asphalt pavement, RAP usually contains chunks of individual aggregates held together by aged binders. As illustrated in Figure 2, RAP pieces can be present in different forms. The size of these chunks depends on milling parameters (depth, machine speed, and blade gap) as well as aggregate toughness, pavement material type, pavement age, and environmental conditions [55,56]. A lower milling speed reduces agglomeration but may increase aggregate breakdown, leading to higher fine content. Therefore, optimizing milling parameters is essential to balance agglomeration and aggregate breakdown.

#### 3.2.1. Types of RAP Agglomeration

Two types of agglomerations occur in recycled asphalt mixes: original agglomeration and new agglomeration. Original agglomerates are bonded RAP aggregates and mortar that cannot be broken by conventional crushing and screening. New agglomerates form during mixing when activated aged binder causes re-bonding of RAP aggregates [46,51]. Fine RAP is particularly prone to agglomeration due to their higher surface area and binder content [57].

Several studies have examined the formation of new agglomerates, suggesting that adhesion between RAP and virgin materials occurs rather than full activation of aged binder. Bressi et al. [46] found that the stiff outer asphalt layer of a RAP particle gradually wears away due to abrasion during, exposing the softer inner binder. This exposed binder then bonds with other RAP particles, generating new agglomerates. Using discrete element method (DEM) simulations, Liu et al. [58] observed that as RAP agglomerates break down, the number of contact bonds within individual clusters decreases, even though the total number of bonds increases, indicating new cluster formation. The rate of bond reduction varies with cluster size, with a critical size of 7.1 mm exhibiting the poorest dispersion, thus requiring more mixing effort.

High mixing temperature and prolonged mixing time increase the wetted surface area of RAP particles, increasing their tendency to bond and form new agglomerates [59]. A temperature threshold likely exists above which the aged binder becomes sufficiently mobilized, allowing for uniform blending with virgin materials and reducing agglomeration risk. Moreover, the quantity and angularity of virgin aggregates influence agglomeration; a higher proportion of angular virgin aggregates enhances abrasion, thus minimizing re-agglomeration [60]. He et al. [61] claimed that a 150% ratio of virgin coarse aggregates to RAP could effectively prevent re-agglomeration.

**Figure 3:**
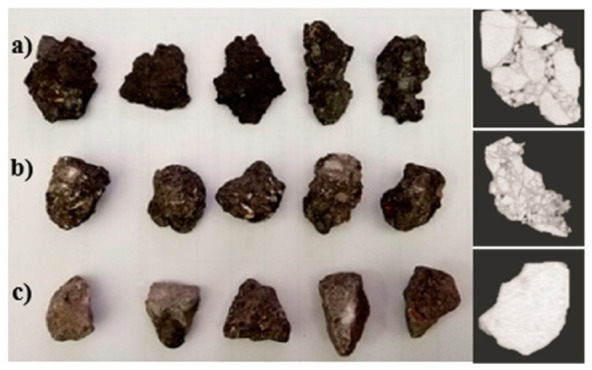
RAP particle forms: (**a**) weak agglomeration; (**b**) strong agglomeration; (**c**) single aggregate (reproduced from [55,64] under the CC BY license, © 2024 The Authors).

In addition, some agglomerates originate from pre-existing agglomerated RAP particles present in milled or minimally processed RAP. Most observed agglomeration in recycled mixes is original [62]. Zhu and Xu classified RAP particles into three forms: individual aggregates, weak agglomerations, and strong agglomerations [63,64]. As shown in Figure 3, weak agglomerations consist of a larger aggregate surrounded by smaller particles and asphalt mortar, which are fragile and tend to break apart during mixing. Strong agglomerations, which occur primarily in fine fractions, are composed of smaller-sized particles bound together within asphalt mastic. Lu et al. [65] defined strong RAP agglomerates as clusters remaining intact during hot mixing. Their proposed degree of separation ratio (DSR) quantifies agglomerate dissociation by comparing gradation changes before and after mixing followed with binder extraction. As particle size decreases, the proportion of strongly bound agglomerates increases, and weakly bound agglomerates tend to decrease.

#### 3.2.2. Quantification of RAP Agglomeration

RAP agglomeration can be identified by micro-spectroscopy and binder tracing methods [51,62,66]. By measuring the aged binder film, Pape et al. [67] demonstrated that variations in film thickness can serve as an indicator of agglomeration degree. Bressi et al. [60] proposed an idealized scenario in which each RAP aggregate is fully separated and uniformly coated with a blended binder of aged and virgin asphalt. The degree of agglomeration can then be quantified by comparing the complex modulus of the blended binder extracted from the real fine fraction versus from the idealized mixtures. However, these methods do not account for changes in aggregate size, which can influence the formation and breakdown of pseudo-sized particles.

Table 2 summarizes various indices used to quantify the degree of RAP agglomeration. Some methods compare the difference in retained weight or passing percentage of each size fraction before and after binder extraction or ignition. In references [19,63,64,68,69], the degree of agglomeration is directly assessed from the loss of retained weight caused by the breakdown of false particles after extraction. However, the weight loss observed on a given sieve includes the portion of weight loss that accumulates on subsequent (smaller) sieve sizes. As a result, this overlapping calculation may exaggerate the agglomeration degree. References [64] (Cantabro test method) and [70] applied the cumulative weight loss to define the agglomeration degree, which is similar to the fineness modulus method [71]. These approaches are suitable for evaluating the overall agglomeration degree but cannot determine the agglomeration type or its severity across different size fractions. Other indices evaluate the overall agglomeration degree by accounting for cumulative changes across all sieve sizes, specifically comparing areas under the gradation curves of the original RAP and the recovered aggregates [61,72,73]. Both approaches have their merits; however, the area-under-the-curve method may be compromised if a specific sieve size is missing due to the non-linear scale of x-axis. Analyzing agglomerated particles within individual size fractions helps identify the size ranges most prone to agglomeration and the dominant agglomeration forms. Therefore, it is recommended to examine the weight loss on each size fraction alongside the overall shift in the gradation distribution.

Tang et al. [71] examined the correlation between RAP agglomeration degree and several key properties, including particle size, asphalt content, and surface area. The results indicated that agglomeration generally decreases as particle size increases. In contrast, Xu et al. [64] observed that agglomeration occurs primarily in coarse RAP. They further reported that coarse aggregates larger than 4.75 mm tend to bond with particles of the next smaller size fraction (including 4.75 mm particles), forming weak agglomerations that contribute to the highest degree of agglomeration and the lowest structural stability. Similarly, Gao et al. [54] investigated the size susceptibility of RAP agglomeration and reported similar results with those of Xu’s study. Their findings showed that each size fraction contains more than 50% of smaller particles, and this percentage increases with the nominal sieve size.

### 3.3. Impact of RAP Variability on Recycled Mix Performance

RAP variability negatively impacts the stability and performance of recycled mixes, prompting specifications to regulate the maximum RAP content [74,75]. In China, technical specification JTG F40-2017 [76] requires the coefficient of variation of RAP binder content to be less than 0.2% to ensure that the asphalt–aggregate ratio of the recycled mix does not deviate by more than 0.3%. For gradation, the standard deviations of the passing percentage should be controlled at 1%, 3%, and 4% for aggregate sizes smaller than 0.075 mm, 0.075 mm to 4.75 mm, and larger than 4.75 mm, respectively. These values are used to estimate the maximum allowable RAP content [76]. In Germany, the maximum allowable RAP content is regulated based on softening point, aged asphalt content, and mass fractions of each aggregate size [77]. In Japan, in addition to controlling asphalt content and the stiffness of RAP binder, the specification requires the processed RAP to contain no more than 5% fines (<0.075 mm) [42].

Agglomeration has been identified as a primary cause of RAP variability. The negative impacts of RAP agglomeration on recycled asphalt mixes include the following:

(1) Reduced binder activation

Aged binder trapped within agglomerated particles cannot fully interact with virgin binders or rejuvenators, hindering binder mobilization and reducing blending homogeneity [78]. Additionally, agglomerates prevent virgin binders from properly coating individual RAP aggregates, leading to insufficient bonding between RAP and virgin materials [66]. To mitigate these issues, it is essential to consider the partial binder contribution when determining the optimal binder content and volumetric properties in mix design [79].

(2) Inconsistent aggregate gradation

The design gradation of recycled asphalt mixes is based on the composite gradation of virgin aggregates and recovered RAP aggregates. However, the incomplete dispersion of RAP particles leads to false particle size distributions as smaller particles agglomerate into larger-sized clusters [80]. Consequently, the actual composite gradation deviates from the designed gradation. This deviation becomes more pronounced with higher RAP content, compromising the structural integrity of recycled asphalt mixes [81].

(3) Inhomogeneous aggregate dispersion and increased air voids

RAP agglomeration disrupts the mix skeleton and impairs aggregate distribution uniformity [82,83]. As RAP content increases, the heterogeneity of RAP aggregates dominates the overall heterogeneity of recycled mixes, leading to poorer aggregate uniformity [84]. This, in turn, increases air void content, reduces compaction efficiency, and causes air entrapment [60]. As a result, water and oxygen can easily penetrate, accelerating oxidation and moisture damage, which further accelerates pavement degradation.

(4) Weakened mechanical performance

The presence of RAP agglomerates creates regions of varying stiffness within the mix, leading to non-uniform mechanical behavior [85]. Weak agglomerates can disintegrate under loading, causing localized structural failures. Conversely, regions with high concentrations of agglomeration can exhibit excessive stiffness, leading to stress concentrations [86]. Yang et al. [47] observed that mixtures containing RAP with a high agglomeration degree exhibited reduced rutting resistance and early stripping in Hamburg Wheel Tracking (HWT) tests, particularly when RAP gradation varied between 9.5 mm and 25 mm. Similarly, Pape and Castorena [62] found that fatigue failure often occurs in the virgin binder matrix surrounding RAP agglomerations.

### 3.4. Summary

This section has examined RAP variability from two interrelated sources: differential binder aging and agglomeration. Differential aging is correlated with particle size, where finer fractions exhibit greater stiffness due to more severe aging. Moreover, differential aging between pavement layers and across particle size fractions leads to uneven binder activation and migration, complicating the accurate determination of virgin asphalt replacement. Agglomeration was identified as a primary driver of RAP heterogeneity. Weak agglomerations tend to break under mixing or loading, while strong agglomerations—predominantly in fine fractions—remain intact and perpetuate false gradation. Although various quantification indices have been developed to measure the degree of agglomeration, each has its merits, and no standardized quantification method has been established due to the complex composition and distinctive size distribution of RAP sourced from multiple locations. The consequences of RAP variability on recycled mix performance include reduced binder activation, inconsistent aggregate gradation, inhomogeneous aggregate dispersion, increased air voids, and weakened mechanical strength.

In summary, addressing RAP variability requires not only accurate characterization of binder aging and agglomeration but also processing strategies that minimize agglomeration and promote uniform dispersion. The quantification methods reviewed in this section provide a foundation for assessing agglomeration; however, a standardized, practical index suitable for routine quality control remains lacking. 

## 4. RAP Processing Techniques

To mitigate the challenges associated with RAP variability, advanced processing techniques have been developed to improve the quality and consistency of RAP materials. These methods aim to minimize agglomeration, enhance binder activation, and ensure uniform gradation, thereby facilitating higher RAP utilization in recycled asphalt mixes. Conventional crushing and screening are the most widely adopted and mandated RAP processing practices, which help reduce RAP variability by enabling stockpiling according to particle size range. Still, this step alone cannot break agglomerated particles or remove aged asphalt mortar adhered to larger aggregates. To overcome these limitations, refined mechanical separation methods have gained significant attention due to their ability to recover RAP aggregates and minimize the residual aged binder content. Unlike solvent-based separation methods, mechanical approaches—such as rotary centrifugal decomposition—are cost effective, involve simpler procedures, and require no additional binder recovery processes.

This section introduces several refined separation methods and compares their advantages and disadvantages. The procedure and benefits of rotary centrifugal decomposition are described in detail to illustrate its potential for refined separation treatment.

### 4.1. Conventional RAP Processing

The most commonly employed RAP processing techniques include crushing, screening, and fractionation, followed by the separate stockpiling of fractionated RAP. These methods aim to reduce particle size and improve material uniformity.

**Figure 4:**
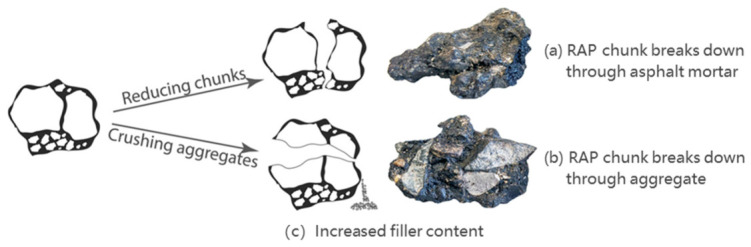
Possible RAP chunk breakdown results (reproduced from [56,57] under the CC BY license, © 2021 The Authors).

Crushing is a primary step used to break down RAP chunks using various types of crushers, such as horizontal-shaft impactors, roller/mill-type breakers, compression-type crushers (e.g., jaw crushers and cone crushers), and hammermill crushers [7]. Figure 4 illustrates the possible results of RAP chunks after crushing. It reduces large chunks to smaller and more uniform particles, improving material consistency. However, a notable drawback is the tendency to increase the fine fraction content in RAP stockpiles. Excessive filler content can elevate the dust-to-binder ratio, reduce mix density, and increase mixture stiffness, particularly in high-RAP-content mixes, thereby adversely affecting volumetric properties and overall pavement performance [49].

Screening and fractionation divide RAP into different size fractions, typically using a size threshold such as 4.75 mm or 2.36 mm [87,88]. The sequence of crushing and sieving can vary depending on material characteristics and processing objectives. For example, pre-screening can minimize aggregate degradation and reduce dust generation. Fractionation often separates RAP into coarse and fine fractions, though some practices recommend dividing into three size ranges: 0–5 mm, 5–10 mm, and 10–15 mm. Although fractionation is not mandatory, it offers greater flexibility in mix design by allowing different size fractions tailored for specific requirements [88].

Traditional RAP processing methods are adapted from equipment and procedures designed for virgin aggregates. However, RAP presents unique challenges due to its heterogeneous composition. Unlike virgin aggregates, RAP particles are coated with aged asphalt binder, which imparts adhesive properties that promote agglomeration. To further improve RAP quality and consistency, refined separation technologies have been introduced to mitigate variability at its source by effectively breaking apart agglomerated particles, enabling more reliable and sustainable reuse of RAP in asphalt production.

### 4.2. Refined Separation Methods

Refined separation aims to recover individual aggregates and mineral fillers. Ideally, this process removes the aged binder film or asphalt mortar from the surface of coarse aggregates, restoring them to a state comparable to virgin aggregates. Several refined separation methods have been developed in research and applied in practice, including chemical separation, bio-degradation separation, mechanical separation, and supercritical separation.

#### 4.2.1. Chemical Separation

**Figure 5:**
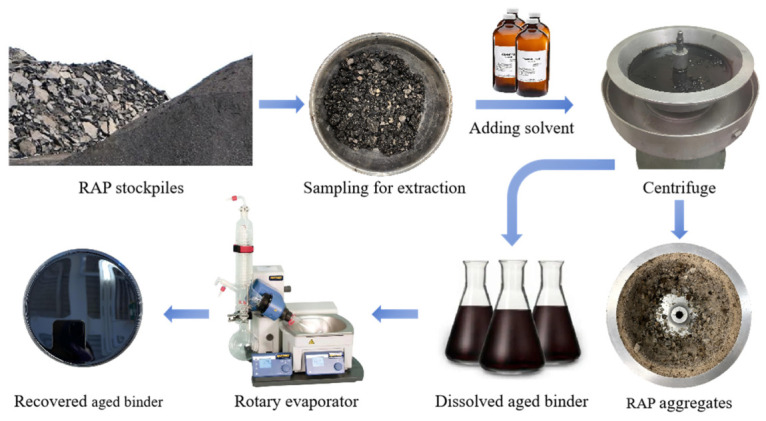
RAP binder extraction and recovery process.

Chemical separation exploits the solubility of asphalt in organic solvents to dissolve the aged binder. As shown in Figure 5, this process involves immersing crushed RAP in solvent, followed by filtration, extraction, and centrifugation to separate the binder from the aggregates [89]. Chemical separation is commonly used in quality control for determining asphalt content and aggregate gradation, as well as for assessing the rheological properties of recovered binders. Standardized procedures exist for centrifugal extraction and evaporation recovery, typically utilizing trichloroethylene, dichloromethane, and toluene as solvents [90].

Although chemical separation is highly effective in completely dissolving the asphalt and preserving aggregate integrity, it is time consuming, requiring multiple filtration and recovery steps with specialized apparatus. Additionally, the recovered binder cannot be directly reused without further treatment. Moreover, most solvents are toxic and volatile, posing environmental and health risks. To make chemical separation viable for plant-scale production, it is essential to develop eco-friendly, economical, and safe operations for recovering aged binder from the extracted solution [18].

#### 4.2.2. Bio-Degradation Separation

Bio-degradation separation offers a more sustainable alternative to chemical methods: it dissolves or degrades the aged asphalt using bio-based solvents or microbial additives. Originating from microbial oil recovery techniques in the petroleum industry, this method employs microorganisms to produce solvents that alter the surface properties of oil reservoir rocks, reducing adhesion between oil and rock [91].

**Figure 6:**
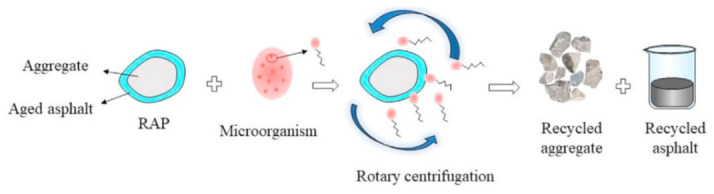
Asphalt–aggregate separation using bio-based degradation method (reused from [18] under the CC BY-NC-ND license, © 2022 The Authors).

In asphalt industry, bio-separation remains in the experimental stage. The goal is to reduce asphalt viscosity, emulsify the mixture, and modify the interfacial wettability between aged asphalt and mineral aggregates, followed by simple centrifugal processing (Figure 6). Despite environmental benefits, bio-separation presents several challenges. Residual surfactants from microorganisms may interfere with the bonding between RAP aggregates and fresh asphalt, and microbial activity in the recovered binder could further alter its chemical and physical properties. Therefore, the selection of bio-solvents must balance efficient separation with compatibility in asphalt.

Liu and Xue developed a bio-separation method using bio-oil to dissolve aged asphalt and simultaneously recover aggregates [92,93,94]. The blend of bio-oil and aged asphalt can serve as a raw binder material, such as liquid asphalt or a binder modifier. Unlike chemical separation, this method uses bio-oil (a rejuvenator) to achieve binder recovery and aggregate extraction at the same time. Nonetheless, concerns remain regarding (1) the solubility of bio-oil at different temperatures, as higher temperatures might be necessary to accelerate dissolution; (2) the inability of recovered blend to be directly reused in asphalt mixes; and (3) the difficulty of separating fine particles from the blended solution, leaving them in an oil sand-like state.

#### 4.2.3. Mechanical Separation

**Figure 7:**
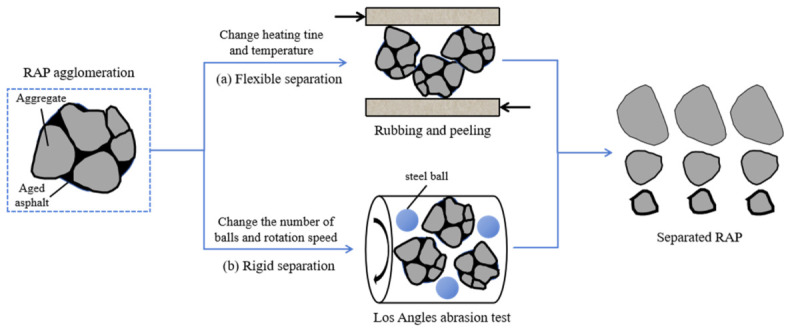
Schematic diagrams of (**a**) flexible separation process and (**b**) rigid separation process.

Mechanical separation employs physical forces—impacting, colliding, squeezing, and rubbing—to remove aged asphalt mortar from the surface of aggregates. Xu et al. [73] proposed two mechanical separation principles: rigid separation and flexible separation. As shown in Figure 7, rigid separation, simulated using a modified Los Angeles abrasion test, involves crashing RAPs against each other and the chamber walls; flexible separation relies on rubbing the heated RAP to generate friction, breaking the adhesion between aged mortar and aggregates.

Mechanical separation is cost effective, easy to assemble, and capable of processing large RAP quantities. The separated coarse aggregates, with residual binder content typically below 1.5%, can potentially be reused as virgin aggregates. Fine fractions, which retain high binder concentrations, require further research to determine viable applications. Despite its advantages, mechanical separation cannot effectively remove aged asphalt from particles smaller than 2.36 mm. Furthermore, it may damage aggregate corners and surface characteristics, and the residual aged asphalt on aggregates can lead to uneven bonding with virgin binders.

**Figure 8:**
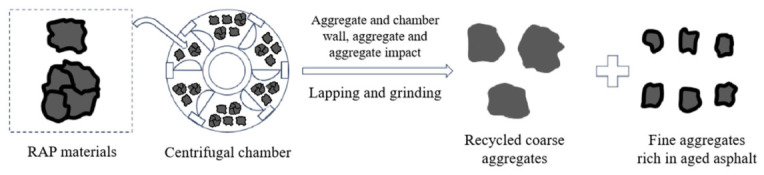
Rotary centrifugal decomposition principle (reproduced from [95] under the CC BY license, © 2025 The Authors).

The ideal separation outcome is to minimize residual aged asphalt while preserving aggregate integrity. Among various refined mechanical separation techniques, rotary centrifugal decomposition has gained prominence. This method utilizes a high-speed rotary device equipped with hard rubber or other specialized materials such as hammerheads and crushing plates (Figure 8). During separation processing, RAP undergoes repeated clapping, crashing, and rubbing, facilitating the removal of brittle aged asphalt mortar from recycled coarse aggregates.

#### 4.2.4. Supercritical Separation

Supercritical separation applies high-temperature and high-pressure water to remove aged asphalt, a technique adapted from hot-water bitumen extraction in the oil sand industry [96]. Derived from the hot water washing method used in oil sand separation, this technique agitates RAP in hot water to break it into individual particles. The rubbing effect removes aged binders and prevents reattachment to aggregate surfaces.

**Figure 9:**
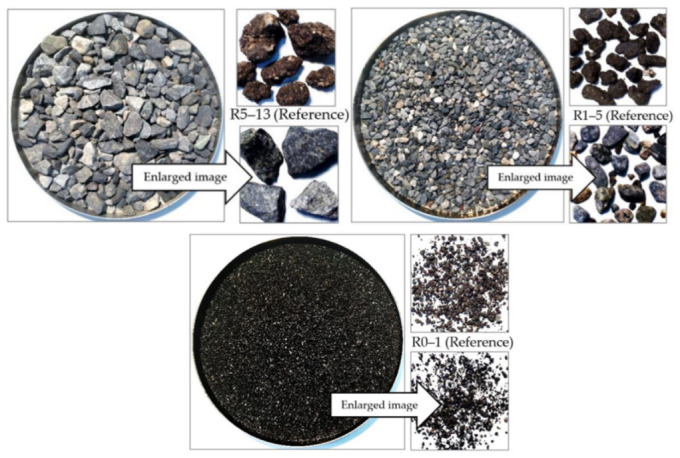
Recovered RAP fractions after hydrolysis separation (reproduced from [97] under the CC BY license, © 2022 The Authors).

In Japan, Akatsu et al. [97,98] developed a hydrothermal separation method that utilizes agitation and stirring in water at 70–90 °C to disaggregate RAP particles. As shown in Figure 9, compared to mechanical separation, this method better preserves aggregate quality, as it involves minimal grinding or crushing. Notwithstanding these advantages, it struggles to recover fine aggregates (<1 mm), and rejuvenating the aged binder requires subcritical water conditions at 300–350 °C and high pressure. These requirements introduce technical challenges, including the need for additional heating systems, thermal recycling infrastructure, and sustainable water management.

#### 4.2.5. Comparison of Separation Methods

Each refined separation method presents distinct advantages and limitations. Chemical separation is most effective but costly and environmentally hazardous. Bio-degradation separation is environmentally friendly but still in experimental stage. Mechanical separation is cost effective but cannot fully remove residual asphalt on aggregates. Bio-oil and hot water washing methods face challenges in recovering aged binders and utilizing the fine aggregates. Table 3 summarizes the working principles, advantages, disadvantages, and current status of the four separation methods discussed above.

When comparing the four technologies quantitatively, rotary centrifugal decomposition can reduce the residual binder on coarse aggregates to less than 2% and offers high processing capacity (80–100 tons per hour) at low economic cost. Although chemical separation still achieves slightly lower residual binder (e.g., <0.5%) and supercritical processing better preserves aggregate quality. Mechanical methods are superior to chemical and supercritical methods in terms of efficiency and binder removal while avoiding toxic solvents or high-pressure requirements. Most important, the processing capacity of rotary decomposition can be improved by increasing the feeding speed and rotational frequency.

The choice of separation method should consider RAP properties, such as asphalt-aggregate adhesion, binder aging level, and weak interface bonding caused by milling. For example, mechanical separation is more suitable for RAP with severely aged asphalt in low-temperature environments, where brittle interface bonding facilitates separation. In terms of efficiency and environmental impact, rotary centrifugal decomposition is the most feasible and economical method.

### 4.3. Rotary Centrifugal Decomposition

Rotary centrifugal decomposition is applied in some regions as a supplementary RAP processing step. This technique utilizes a high-speed rotating decomposition chamber to break RAP agglomerates and remove aged asphalt mortar from coarse aggregates, facilitating the recovery of high-quality reclaimed materials.

In the Netherlands, the Low Emission2 Asphalt Pavement (LE2AP) project applied a rotary decomposition device to separate milled porous asphalt pavement materials into reclaimed aggregates with minimal binder content and bitumen-rich mortar sand [99]. The aged asphalt mortar exhibits glass-like behavior at high frequencies; therefore, it can be easily peeled off from coarse aggregates during repeated impact and collision. The separated mortar sand was subsequently blended with a rejuvenator and soft binder to produce a high-quality asphalt mortar. This rejuvenated mortar was then foam-mixed with preheated reclaimed stones to obtain a porous asphalt mix comprising 93% reclaimed materials, achieving horizontal RAP recycling [100].

**Figure 10:**
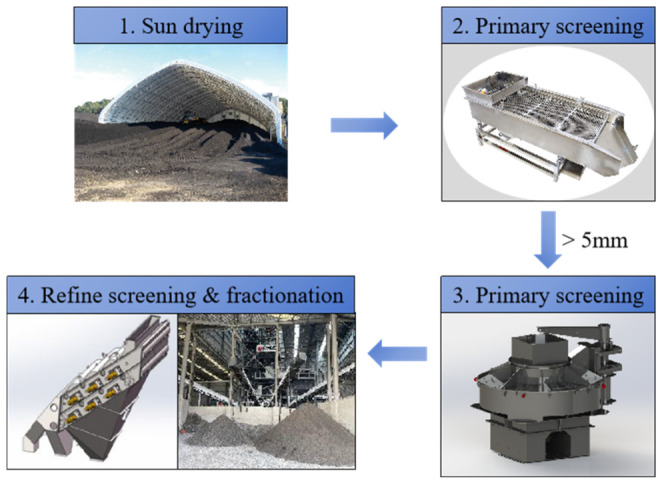
Four main steps in refined RAP separation(reproduced based on [19,95] under the CC BY license).

In China, refined mechanical separation has been increasingly adopted by contractors and agencies for RAP processing. Yu and Li [19,68] described a refined separation procedure comprising four steps (Figure 10):

(1) Sun drying: Natural sun drying reduces moisture content, preventing fine particle agglomeration caused by high moisture levels.
(2) Primary screening: A flip-flow vibrating screen with a 5 mm mesh separates coarse RAP (>5 mm) from fine RAP (<5 mm). Coarse RAP is sent for further decomposition, while fine RAP can be stored for direct reuse.
(3) Asphalt–aggregate decomposition: This step is accomplished in a centrifugal decomposition device, which consists of a vertical spindle, rotator, and crushing chamber.
(4) Refined screening: This step uses a combined sieving system consisting of high-frequency vibrating screens, relaxation sieves, and probability sieves to further separate RAP into three to four size fractions.

**Figure 11:**
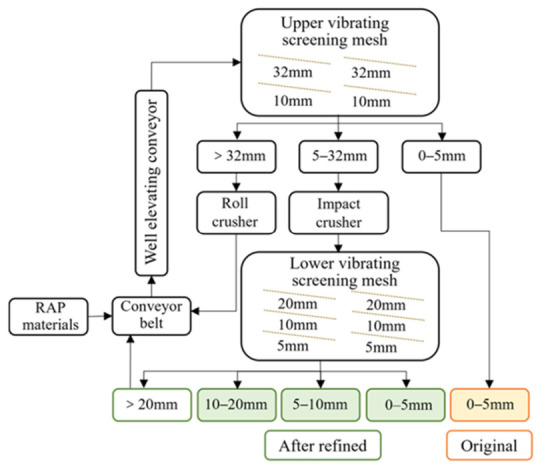
Schematic flowchart of the refined separation procedure (reused from [70] under the CC BY license, © 2024 The Authors).

Wang et al. [70] categorized the separation system into several subsystems, including the feeding system, screening system, impact crusher (i.e., centrifugal decomposition device), roll crusher, dust removal system, and discharging system (Figure 11). The centrifugal decomposition device is the core of this process. Inside the chamber, RAP particles collide and rub against baffles, inner walls, and other particles, effectively stripping aged asphalt mortar from coarse aggregates. Qu et al. [69] enhanced this process by designing the centrifugal chamber with internal wire brushes to more effectively scrape aged asphalt from aggregate surfaces. There are two sources of separated asphalt mortar: one from the primary screening and the other collected after centrifugal decomposition and screening. The latter may contain both asphalt mortar stripped from coarse aggregates and broken edges of recovered coarse aggregates. This phenomenon can increase the inconsistency of separated fine RAP due to its multiple sources and complex composition.

As mentioned above, rotary centrifugal decomposition offers high separation efficiency and processing capacity. However, a balanced assessment requires detailed examination of its limitations and the challenges associated with in situ implementation.

- Equipment maintenance: The high rotational speeds and abrasive nature of reclaimed asphalt pavement (RAP) cause wear on rotor blades, liner plates, and screen surfaces. Liner replacement intervals contribute significantly to operating costs. Additionally, dust generation requires robust collection systems.
- Processing costs and energy consumption: While energy costs are moderate, total operating costs include wear parts, maintenance labor, dust control, and management of the separated fine fraction. Compared to chemical separation (high solvent cost and low throughput) and supercritical processing (high-pressure equipment and limited capacity), rotary centrifugal decomposition offers the best economic return for medium- to large-volume RAP recycling.
- Scalability and field adoption: Although rotary centrifugal equipment has been widely adopted in RAP recycling plants, detailed operational data—such as uptime, wear part life, and product quality variability—remain largely proprietary and not standardized.
- Industrial implementation: The technology is inherently modular. Increasing total plant capacity is achieved by adding parallel units rather than scaling up a single rotor. Future developments may include variable frequency drives to optimize rotor speed for different RAP feedstocks.

### 4.4. Summary

This section has reviewed conventional and refined RAP processing techniques aimed at reducing variability and improving material quality for high-RAP recycling. Conventional crushing and screening are widely adopted but cannot break agglomerated particles or remove aged asphalt mortar. Among the refined separation methods, mechanical separation is most suitable for plant-scale use, as it avoids the challenges of extracting fine fractions from residual solutions. Rotary centrifugal decomposition, in particular, emerges as the most feasible and economical approach, capable of reducing residual binder on coarse aggregates to below 1.5%. However, mechanical separation remains ineffective for particles smaller than 2.36 mm, and the fine fraction—enriched in aged mortar—requires further treatment. Moreover, the separated fine RAP may originate from both primary screening and stripped mortar, introducing additional compositional complexity.

In summary, rotary centrifugal decomposition is a viable supplementary step to upgrade RAP quality, enabling higher RAP content in surface layers and horizontal recycling. Future efforts should focus on optimizing mechanical separation parameters to minimize aggregate damage, developing effective reuse strategies for the fine separated fraction, and establishing quality control protocols for the processed materials.

## 5. Impact of Refined RAP Separation

The most significant improvement observed in separated RAP is the substantial reduction in agglomeration. Beyond this, refined separation treatment also affects the physical properties of RAP aggregates, RAP gradation, and residual binder content. Additionally, the influence of refined separation on the performance of high-RAP-content mixes requires further investigation to validate the necessity of incorporating this extra step into current RAP processing practices.

### 5.1. Properties of Separated RAP

#### 5.1.1. Physical Properties of RAP Aggregates

Once agglomerated particles are separated and the aged asphalt mortar is removed, RAP particles continue to undergo collisions and friction during centrifugal decomposition. Consequently, the reclaimed aggregates do not fully revert to their original state, as seen in virgin aggregates. The physical properties of RAP aggregates are altered, and some residual aged asphalt may remain on their surfaces. Three fundamental indices are used to evaluate aggregate morphological characteristics: sphericity, roughness, and shape factor. These parameters can be assessed using X-ray computed tomography (CT) scanning to obtain the three-dimensional structure of aggregates [101]. Higher values of sphericity and shape factor indicate a more cubic particle shape and reduced angularity.

Wang et al. [70] investigated the effect of rotating frequency on RAP properties during centrifugal decomposition. As frequency increased, the agglomeration degree and asphalt content variability decreased significantly; however, the angularity, crushing value, and elongated/flat particle content of coarse aggregates declined. Yu et al. [19] reported slightly decreased angularity of RAP aggregates, while sphericity and surface texture remained mostly unchanged. Contrary to Wang’s findings, they observed a slight strength (i.e., crushing value) increase due to the removal of weak edges and corners.

Qu et al. [69] evaluated the effects of rotation speed, duration, and RAP temperature on aggregate properties. Rotation speed had the most significant impact on morphological characteristics, followed by duration and temperature. As rotation speed increased, the angularity of coarse aggregates gradually decreased, while sphericity increased until it became less sensitive to further processing. Aggregates larger than 9.5 mm experienced fragmentation, creating new edges and corners; in contrast, smaller particles underwent grinding, resulting in smoother surfaces and higher sphericity.

Wang et al. [102] applied CT scanning and 3D reconstruction to examine the mesoscale morphological characteristics (shape, angularity, and texture roughness) and internal crack damage of reclaimed coarse aggregates (>5 mm). Compared to conventional processing, refined separation reduced the damage rate and increased the surface roughness of coarse aggregates. However, rotation frequency must be controlled to limit the loss of angularity, which—together with texture roughness—was strongly correlated with the crushing value and weight loss percentage.

#### 5.1.2. RAP Gradation

By breaking the agglomerations apart, refined separation eliminates the false particle within RAP, thereby improving its gradation consistency [103,104]. Zhang et al. [95] reported that refined separation significantly reduced the residual deviation values of RAP at several key sieve sizes compared to traditional double-roller crushing, indicating a decrease in false particle content. Consequently, gradation variability was controlled to below 5% for each sieve size fraction (except <0.075 mm). However, excessive separation treatment may impose a refinement effect on RAP gradation.

RAP is inevitably fragmented during milling, leading to a refined gradation; while in rotary centrifugal decomposition, grinding and collisions might further refine the gradation. Li et al. [68] found that refined separation significantly increased RAP refinement level, with the particle refinement index for fractions below 4.75 mm reaching 30% to 50%. In some cases, the fine powder content (<0.6 mm) can constitute up to 77% of the separated fine fraction (0–3 mm) [69]. Xu et al. [73] reported that mechanical separation could result in excessive pulverization and gradation refinement for 5–10 mm RAP aggregates due to increasing impact energy as separation progressed.

#### 5.1.3. RAP Binder Content

**Figure 12:**
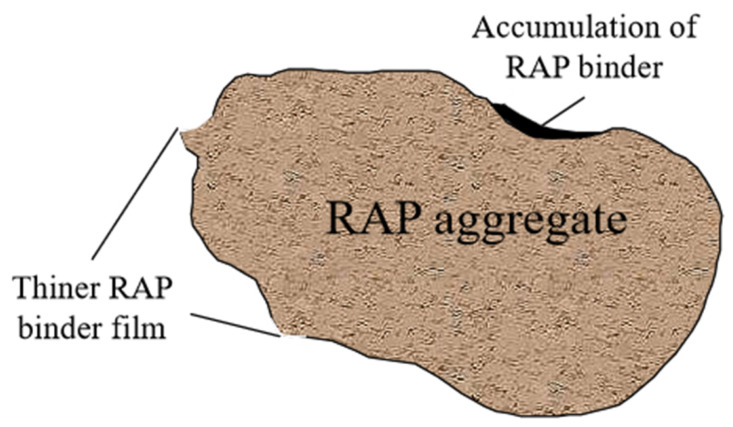
Uneven binder film thickness on a RAP aggregate (reproduced based on [105]).

Another notable effect of refined separation is the reduction of asphalt content in coarse RAP. As shown in Table 4, compared to traditional crushing and screening, asphalt content in decomposed RAP fractions decreases significantly as particle size increases. For RAP particles larger than 5 mm, the asphalt–aggregate ratio drops below 2%. This reduction minimizes blue smoke emissions and asphalt adhesion to mixing cylinder walls when RAP contacts flames or superheated virgin aggregates during hot mixing. However, due to the uneven coverage of aged binder film on RAP aggregates (Figure 12), the residual aged asphalt may interfere with the bonding between the reclaimed asphalt pavement (RAP) and new asphalt [105]. RAP aggregates partially or fully coated with aged binder film prevent the new asphalt from adhering to the aggregate surface and typically form a composite layer [106]. The thickness of the aged film also plays a critical role. Although the film may improve bond strength under dry conditions, an excessively thick layer (>200 µm) can weaken the cohesion strength of the binder and lead to re-agglomeration [107].

Nevertheless, binder content in fine fractions varied from 4.5% to 14% due to different size thresholds and complex composition. The bitumen-rich fraction still needs separate treatment in hot-mix recycling, as direct exposure to a drum burner flame could cause emissions and possible re-agglomeration.

### 5.2. Performance of Recycled Asphalt Mixes Containing Separated RAP

Refined separation improves aggregate dispersion uniformity by reducing RAP agglomeration. Tang et al. applied digital image analysis to evaluate RAP aggregate dispersion in recycled asphalt mixes [83,84]. Their findings indicated that finely separated RAP disperses more easily than traditionally processed RAP, as it contains fewer agglomerates and less adhesive binder. Xu et al. [73] found that laboratory separation reduced the porosity of recycled asphalt mixes, suggesting that RAP aggregates dispersed as individual particles, allowing for better virgin binder coverage.

Regarding pavement performance, recycled asphalt mixes incorporating finely separated RAP exhibit improved low-temperature performance, moisture damage resistance, and fatigue resistance compared to those prepared with untreated RAP [94,109,110]. However, rutting resistance may decrease due to a lower proportion of aged asphalt in separated coarse RAP [47]. Qu et al. [69] reported that asphalt mixes containing separated RAP demonstrated superior fatigue performance compared to traditional recycled mixes due to aged asphalt removal and lower variability in reclaimed aggregates.

### 5.3. Environmental and Economic Implications of Refined Separation

The environmental superiority of rotary centrifugal decomposition over traditional processing methods are demonstrated across multiple quantifiable dimensions. Life-cycle Assessment (LCA) studies demonstrate that recycled HMA incorporating RAP processed by rotary decomposition shows considerably lower environmental impacts than both virgin HMA and traditionally recycled HMA [111]. Specifically, for SMA-13, AC-16, and AC-20 asphalt mixes, the recycled HMA achieves reductions in energy consumption and CO_2_ emissions of 5%, 10%, and 15%, respectively. In terms of waste reduction, the refined decomposition process allows for higher RAP incorporation rates without sacrificing performance. Conventional processing methods typically restrict RAP content to 15–20% because of agglomeration-related variability and performance loss. In contrast, rotary centrifugal decomposition permits RAP content as high as 70% while still meeting or exceeding the performance of conventional mixtures [95].

Refined separation also provides direct economic benefits through reduced total project costs. By employing centrifugal impact or advanced fractionation, refined processing liberates aggregates to a state where residual binder content can drop below 2%, making them directly substitutable for virgin materials [109]. In a high-proportion plant-produced hot-recycled asphalt mixture, refined-separation recycled asphalt pavement lowers total cost by 33% relative to conventional crushed and screened RAP, and by 50% relative to virgin asphalt pavement [112]. In addition to direct material substitution, refined processing generates indirect economic advantages by enhancing mixture workability and extending pavement service life. Improved workability reduces plant energy consumption and compaction effort during paving. For instance, finely separated RAP demonstrate a 13.35% improvement in low-temperature stability, a 1.28% increase in residual stability, and a 5.39% enhancement in freeze–thaw splitting strength when compared to conventional recycled mixtures [113].

### 5.4. Potential Limitations

Refined separation techniques, such as rotary centrifugal decomposition, are beneficial in reducing agglomeration and improving material consistency, but they also introduce challenges that must be addressed to ensure practical and sustainable applications.

- Damaged aggregate properties: High-intensity separation treatment can negatively impact the physical properties of RAP aggregates. Excessive grinding and mechanical forces will reduce the angularity and toughness of separated coarse aggregates, potentially affecting mechanical interlock and stability of the final recycled asphalt mix. Parameters such as rotation speed, separation duration, and temperature must be controlled to balance aged asphalt removal while preserving aggregate integrity.
- Gradation refinement of coarse RAP: Refined mechanical separation can cause unintended over-refinement of coarse RAP. Excessive fragmentation generates an undesirable gradation with too much fine content, reducing the availability of RAP needed for certain asphalt mix designs. It is essential to maintain the balance between separation efficiency and gradation control.
- Recycling separated fine RAP particles: Mechanical separation produces a significant amount of fine RAP (0–3 mm), which can account for approximately 40% of processed RAP. This fraction includes mineral filler, natural sand, and asphalt powder, making it difficult to reintegrate into large-scale asphalt production. The ultra-high bitumen content may lead to issues such as excessive binder stiffness or reduced workability.
- Increased virgin asphalt demand: Refined separation removes aged asphalt and bitumen-rich mortar from coarse RAP aggregates, thus reducing the overall binder content. Consequently, higher amounts of virgin asphalt are required, especially in mixes containing high RAP percentages or fully reclaimed aggregates. If aged binder is not effectively recovered or rejuvenated, material costs may rise due to the higher unit price of virgin asphalt.

## 6. Conclusions and Recommendations

The findings of this review highlight the critical role of refined separation treatment in reducing the variability of RAP materials, thereby promoting better performance consistency and a higher recycling rate. Mechanical separation remains the most practical method for large-scale RAP processing, and advanced techniques such as rotary centrifugal decomposition show great potential for achieving high-quality separated RAP. However, implementing refined separation on a broader scale depends on factors such as cost effectiveness, processing efficiency, aggregate degradation control, and the long-term performance of asphalt mixes containing separated RAP. The main conclusions and recommendations for future research drawn from this review are as follows:

### 6.1. Conclusions

- RAP agglomeration is the primary contributor to variability, causing false particle gradation, inaccurate binder contribution, and non-uniform aggregate distribution in recycled asphalt mixes. Refined separation techniques, particularly rotary centrifugal decomposition, effectively reduce agglomeration and improve material homogeneity.
- Among different refined methods, mechanical separation is most feasible for large-scale application. Other methods—such as chemical and bio-degradation approaches—remain at laboratory scale due to their complexity, time-consuming procedures, and environmental concerns. Rotary centrifugal decomposition has proven capable of producing high-quality reclaimed coarse aggregates with residual binder content below 2%, approaching virgin aggregate quality. However, this technology imposes repeated impacts on RAP that can reduce aggregate angularity and generate additional fines.
- The bitumen-rich fine fraction (0–5 mm), which can constitute over 40% of separated RAP and has a high asphalt-aggregate ratio exceeding 10%, presents both a challenge and an opportunity. While coarse RAP can be effectively recovered and reused, the fine fraction poses unique challenges due to its high surface area, agglomeration tendency, and variability in binder aging. If properly homogenized and regenerated, it could significantly reduce the demand for virgin asphalt binder.

### 6.2. Recommendations for Future Research

- Long-term durability validation: Further investigation is needed on the impact of refined separation treatment on RAP incorporation rates and mix performance, particularly long-term durability of mixes containing high RAP content (up to 100%). Accelerated pavement testing (APT) can simulate 10–20 years of traffic in months, and key performance indicators include rut depth, cracking density, ride quality, and frictional resistance should be monitored. Special attention should be given to coarse aggregate characteristics and the influence of residual aged asphalt film on bonding performance.
- Rejuvenation for bitumen-rich fines: Research should develop cost-effective rejuvenation protocols for the fine fraction. However, uniform dispersion is difficult because the fine particles tend to clump, causing localized softening. Advanced mixing techniques (e.g., high-shear blending or pre-treatment with warm-mix additives) are under investigation to improve homogeneity of the rejuvenated mortar.
- Alternative application of separated fine RAP: Identifying feasible, high-value applications beyond conventional asphalt mixes for separated fine RAP is important. Given the presence of severely aged asphalt and high filler content, alternative treatment or fabrication methods may be needed for specialized uses such as micro-surfacing, stress absorption layers, and cracking sealing materials in pavement construction and maintenance.
- Life-cycle cost analysis: Future studies should evaluate the economic feasibility of refined separation techniques. Key questions include whether the additional costs of equipment development, installation, extra storage space, and multiple stockpile management are justified compared to established techniques. Research should also determine whether the increased value of separated RAP can offset the time and energy costs associated with this extra processing step.
- Parameter optimization and standardization: Systematic experimental studies are required to optimize rotary centrifugal decomposition parameters (e.g., rotation frequency, duration, and temperature) to balance aged asphalt removal, aggregate integrity, and gradation control while minimizing the production of excessive fines. Response surface methodology or evolutionary algorithms could identify Pareto-optimal operating windows. Ideally, standardized guidelines and quality specifications should be proposed on maximum residual binder content, acceptable fine fraction limits, and performance criteria to enable wider adoption of this technique.
- Emerging research opportunities: Future work should also explore (a) artificial intelligence (AI) and machine learning for real-time RAP quality prediction using near-infrared spectroscopy or image analysis to adapt separation parameters dynamically; (b) advanced characterization of agglomeration mechanisms via X-ray micro-CT and surface energy measurements; (c) integration of separation with in-line rejuvenation for one-step processing; and (d) material circularity indicators tailored to RAP separation technologies.

## Figures and Tables

**Table 1:** Virgin asphalt grade selection in recycled hot-mix asphalt [39].

Penetration of RAP Binder (0.1 mm at 25 °C)	RAP Content (%)	Selection of Virgin Asphalt
*p* ≥ 30	<20%	No change
	20~30%	Bump up 1 mm of P@25 °C
	≥30%	Based on the blending chart
20 ≤ *p* < 30	<15%	No change
	15~25%	Bump up 1 mm of P@25 °C
	≥25%	Based on the blending chart
10 ≤ *p* < 20	<10%	No change
	10~15%	Bump up 1 mm of P@25 °C
	≥15%	Based on the blending chart

**Table 2:** Summary of RAP agglomeration quantification indices.

Reference	Test Methods	Agglomeration Degree Indices	Principle	Explanation
[19]	Sieving and extraction	$WLP=\frac{\left(W-W_{R}-W_{A}\right)}{W}$ - W is the RAP weight before extraction. - $W_{R}$ is the RAP weight retained on the original sieve after extraction. - $W_{A}$ is the weight of aged asphalt.	Retained weight loss	A higher weight loss percentage (WLP) indicates a greater agglomeration degree.
[59]	Mixing and sieving	$CDI=\frac{W_{i}-W_{f}}{W_{i}}\times 100$ $AI=\frac{W_{f(i+1)}}{W_{i}}\times 100$ - $W_{i}$ is the mass of RAP retained on ith sieve before heating and mixing. - $W_{f}$ and $W_{f(i+1)}$ are the masses of RAP retained on ith sieve and one size larger after heating and mixing.	Retained weight loss	The cluster dissociation index (CDI) measures the extent of RAP cluster dispersion during hot mixing, while the agglomeration index (AI) quantifies the formation of re-agglomerated particles.
[61]	Sieving and extraction	$OAR=\frac{{Area}_{ER}-{Area}_{BR}}{{Area}_{ER}}\times 100\%$ - ${Area}_{ER}$ and ${Area}_{BR}$ are the areas under the gradation curves of extracted RAP aggregates and as-received RAP after milling, respectively.	Overall gradation shift	The original agglomeration rate (OAR) represents the degree of agglomeration in RAP immediately after milling.
[63]	Sieving and extraction	${Loss\%}_{extraction}=\left(1-\frac{w_{retained}}{w}\right)\times 100\%$ - w is the weight of the RAP before extraction. - $w_{retained}$ is the weight of extracted RAP aggregates retained on the original sieve size.	Retained weight loss	The loss percentage (Loss%) reflects the agglomeration degree of RAP, with higher values corresponding to a greater degree of agglomeration.
[64]	Sieving and extraction	${PLI}_{i}=\left(1-P_{i}\right)\times 100\%$ - $P_{i}$ is the percentage of RAP retained on each sieve size.	Retained weight loss	The percentage loss index (PLI) quantifies the degree of RAP agglomeration at each size fraction.
[64]	Cantabro crushing test and extraction	$w=\frac{1}{n}\sum_{i=1}^{n}\left(\frac{S_{c,i}-S_{i}}{S_{c,i}}\right)\times 100\%$ - n is the number of RAP fraction sizes. - $S_{c,i}$ and $S_{i}$ are the loss rates of ith sieve size after extraction and after crushing, respectively.	Accumulated retained weight loss	The index of stability (w) quantifies the overall clustering stability of RAP, with higher values indicating greater resistance to the breakdown of agglomerated RAP particles.
[68]	Sieving and extraction	$L=P_{RAP-aggregate}-P_{RAP}$ - $P_{RAP-aggregate}$ is the passing rate of extracted aggregates at the lower sieve size. - $P_{RAP}$ is the passing rate of RAP at the lower sieve size, which is 0%.	Retained weight loss	The passing rate of the lower sieve size after extraction is used to quantify the degree of agglomeration within a specific size range (e.g., 13.2 mm–16 mm).
[69]	Sieving and ignition	$\rho =\frac{W_{1}-W_{2}-(m_{0}-m_{1})}{W_{1}}\times 100$ - $W_{1}$ and $W_{2}$ are the masses of RAP retained on a given sieve before and after separation. - $m_{0}$ and $m_{1}$ are the masses of RAP before and after ignition.	Retained weight loss	The passing rate difference of pre-screened coarse RAP at the lower sieve size within a given range (e.g., 9.5 mm for 9.5–16 mm RAP) is used to assess agglomeration.
[70]	Sieving and extraction	$I_{C}=\frac{\sum_{i=1}^{n}\left|P_{i}^{b}-P_{i}^{a}\right|}{n}$ - $P_{i}^{b}$ is the passing percentage of ith size before extraction. - $P_{i}^{a}$ is the passing percentage of ith size after extraction.	Accumulated retained weight loss	The agglomeration degree is determined based on the accumulated difference in sieve residue before and after the extraction.
[71]	Sieving and extraction	$f=\frac{\left(U_{2}+U_{3}+\cdots +U_{i-1}+U_{i}\right)-\left(i-1\right)\times U_{1}}{\left(100-U_{1}\right)}$ $FMR=f_{AGG}/f_{RAP}$ - f is the fineness modulus. - $f_{AGG}$ and $f_{RAP}$ are the fineness moduli of recovered RAP aggregates and the original RAP. - $U_{1}$ to $U_{i}$ are the cumulative retained percentages from nominal maximum aggregate size to 0.075 mm.	Overall gradation shift	The fineness modulus ratio (FMR) reflects the overall agglomeration degree of RAP, with higher FMR values indicating a lower degree of agglomeration.
[72,73]	Sieving and extraction	$C=\frac{S_{before}-S_{overlap}}{S_{after}}$ - $S_{before}$ and $S_{after}$ are the areas under the percentage retained curves of RAP before and after extraction. - $S_{overlap}$ is the overlapped area by the abovementioned two curves.	Overall gradation shift	The agglomeration index (C) ranges from 0 to 1, with values closer to 1 indicating a higher degree of agglomeration.

**Table 3:** Comparison of refined RAP separation methods.

Method	Principle	Residual Binder on Coarse RAP (%)	Aggregate Degradation	Processing Capacity	Pros and Cons	Current Status
Chemical	Solvent dissolution	<0.5	Very low	less than one batch	Complete binder removal; utilization of toxic, volatile solvents; time intensive.	Widely used in QC/lab; not practical for plant scale
Bio-degradation	Microbial or bio-solvent degradation	3–3.5	Low	experimental scale	Sustainable; simultaneous binder rejuvenation; residual surfactants may affect bonding; microbial activity may change binder properties	Research/lab only
Mechanical (rotary decomposition)	Collision and scrubbing	<2	Moderate	80–100 tons per hour	Time efficient; accessible recovered coarse aggregates; potential surface abrasion and loss of aggregate angularity	Applied in practice
Supercritical	Hydrothermal agitation at high pressure	<1	Very low	experimental scale	Preserves aggregate quality due to minimal abrasion; non-recoverable fines; high energy and pressure demand	Pilot/limited application (Japan)

**Table 4:** RAP fractions and binder content treated with different separation methods.

Reference	Separation Method	Products After Separation	Size Range (mm)	Asphalt Content (%)
[108]	Fractionation	Coarse RAP	4.75–13	3.7
		Fine RAP	<4.75	5.8
[69]	Fractionation	Coarse RAP	9.5–16	2.9
		Coarse RAP	4.75–9.5	3.3
		Fine RAP	2.36–4.75	4.4
		Fine RAP	<2.36	8.4
[68]	Crushing and screening	Coarse RAP	12–22	5.0
		Coarse RAP	5–12	5.5
		Fine RAP	<5	6.8
[97]	Hydrothermal rubbing	Recovered aggregates	5–13 and 1–5	0–0.9
		Fine particles	<1	Unknown
[109]	Centrifugal decomposition (shot blasting machine)	Coarse RAP	10–20	1.5
		Coarse RAP	5–10	1.5
		Fine RAP	<5	>10.0
[68]	Centrifugal decomposition	Coarse RAP	10–15	2.2
		Coarse RAP	5–10	2.44
		Fine RAP	<5	6.5 and 6.7
		Recycled asphalt powder	<0.075	14.8
[69]	Centrifugal decomposition	Separated RAP	9.5–16	0.66
		Separated RAP	4.75–9.5	0.85
		Separated RAP	2.36–4.75	0.91
		Bitumen-rich mortar	<2.36	10.0–14.0
[70]	Centrifugal decomposition	Coarse RAP	10–20	0.9–2.0
		Coarse RAP	5–10	1.1–2.5
		Fine RAP	<5	6.5–8.0
[100]	Centrifugal decomposition	Coarse aggregates	5–8 and 8–16	1.0
		Bitumen-rich mortar	<2	10.0–14.0
[110]	Centrifugal decomposition	Coarse RAP	10–15	1.2
		Coarse RAP	5–10	2.2
		Fine RAP	<5	4.8

## Data Availability

No new data were created or analyzed in this study. Data sharing is not applicable to this article.
